# Phase 1b trial on the safety, tolerability and immunogenicity of anti‐amyloid vaccine ALZ‐101 in subjects with MCI or mild AD

**DOI:** 10.1002/alz.095440

**Published:** 2025-01-09

**Authors:** Henrik Zetterberg, Juha O. Rinne, Anders Sandberg, Zsófia Lovró, Mika Scheinin, Stefan Pierrou, Kirsten Harting

**Affiliations:** ^1^ Institute of Neuroscience and Physiology, University of Gothenburg, Gothenburg, Mölndal Sweden; ^2^ Turku University Hospital, Turku Finland; ^3^ Alzinova AB, Mölndal Sweden; ^4^ Clinical Research Services, Turku Finland

## Abstract

**Background:**

ALZ‐101 is a vaccine comprised of stabilised oligomeric Aβ42 that stimulates a humoral immune response primarily targeting a toxic, low‐abundant oligomeric form of Aβ. Part A of a clinical Phase 1b trial (ALZ‐C‐001; NCT05328115) was recently concluded with the objectives to assess the safety, tolerability and immunogenicity of ALZ‐101 in subjects with mild AD or MCI due to AD.

**Methods:**

Participants were randomised to receive placebo (n = 6), 125 µg (n = 10) or 250 µg (n = 10) doses of ALZ‐101 at weeks 0, 4, 8, and 16. Primary endpoint safety parameters, including brain MRI scans, were assessed at baseline and before each injection with a follow‐up assessment at week 30. Secondary endpoints included immunogenicity assessments for which blood samples were collected throughout the study. Exploratory endpoints included measuring CSF levels of Aβ, tau, NFL, neurogranin, and inflammation biomarkers for which samples were collected at weeks 0 and 20. Part A of the study includes all data up to week 30; brain MRI was not carried out on all participants at this time point.

**Results:**

26 eligible participants were dosed in the randomised, double‐blind, placebo‐controlled Part A of study ALZ‐C‐001. The average MMSE and CDR‐SB scores at baseline were 23.9±0.6 and 2.9±1.1, respectively.

The study met its primary and secondary endpoints. The vaccine was well tolerated. There were no cases of suspected meningoencephalitis or ARIA‐E. One participant had an MRI finding suggestive of an increase in size of a pre‐existing microhaemorrhage.

The vaccine proved immunogenic with oligomer‐specific antibody titres evolving after three administrations. Th2 activity was confirmed by antigen‐recall assays on PBMCs using ELISpot. Higher antibody titres at week 20 correlated with decreases in several CSF biomarkers of AD (p‐tau181, total tau, and neurogranin), but no such associations were observed for neuroinflammation markers (YKL‐40 and sTREM‐2).

**Conclusion:**

The results suggest that ALZ‐101 vaccination is safe and generates an Aβ oligomer‐specific humoral immune response that has an impact on several biomarkers associated with disease progression. Taken together, these findings support further development of ALZ‐101 as a potential novel disease‐modifying treatment for patients with AD.

